# Supplementary material for: KSHV infection of B cells primes protective T cell responses in humanized mice
Source: Nat Commun. 2024 Jun 6;15:4841. doi: 10.1038/s41467-024-49209-w (PMC11156630; doi:10.1038/s41467-024-49209-w)
Supplement: Supplementary file 1 — Supplementary Information [file 41467_2024_49209_MOESM1_ESM.pdf]

KSHV Infection of B Cells Primes Protective T Cell Responses in Humanized Mice

Nicole Caduff, Lisa Rieble, Michelle Böni, Donal McHugh, Romin Roshan, Wendell Miley, Nazzarena Labo, Sumanta Barman, Matthew Trivett, Douwe M.T. Bosma, Julia Rühl, Norbert Goebels, Denise Whitby and Christian Münz

SUPPLEMENTARY FIGURES

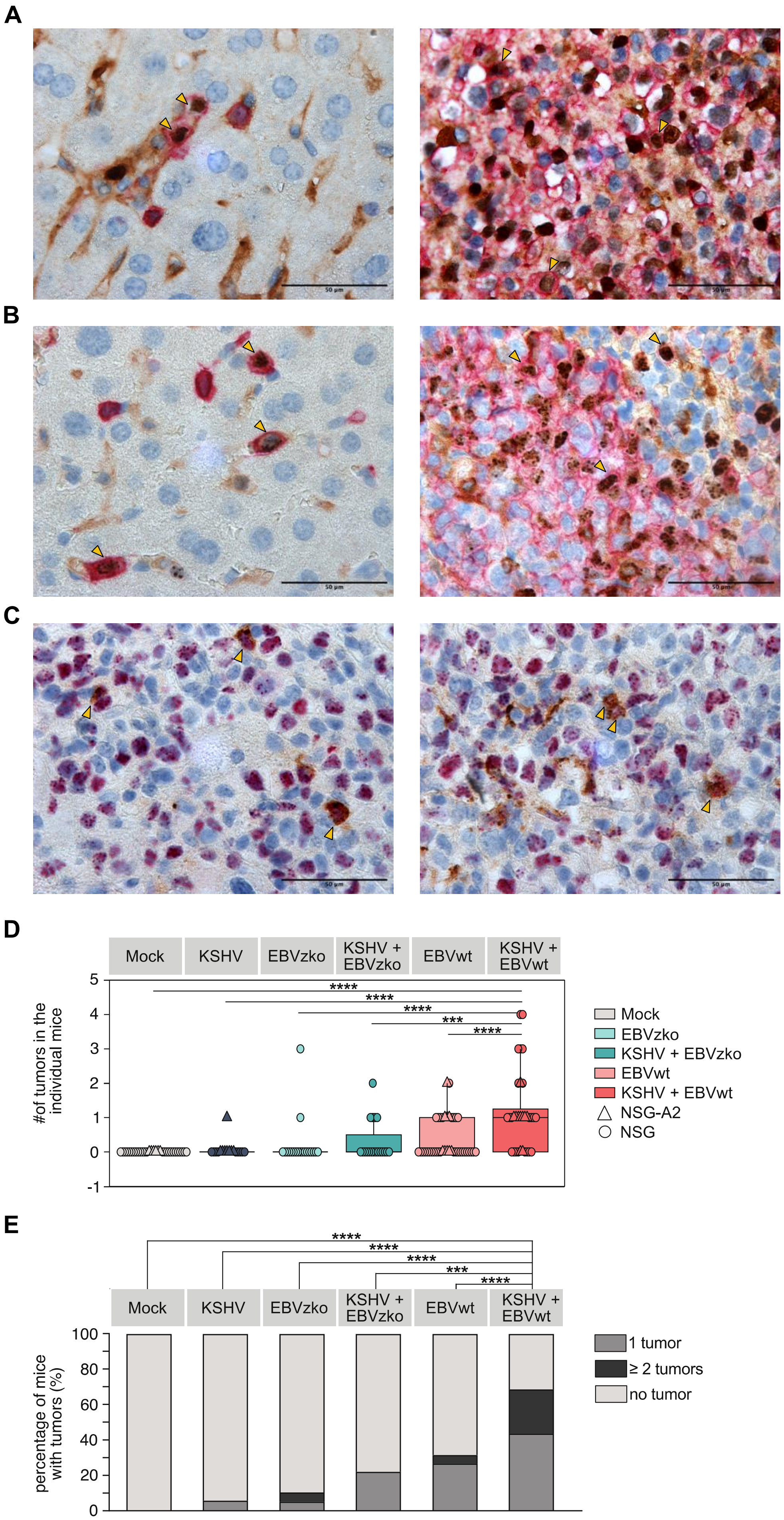
A
B
C
D
Mock
KSHV
EBVzko
KSHV + EBVzko
EBVwt
KSHV + EBVwt
#of tumors in the individual mice
5
4
3
2
1
0
-1
****
****
****
***
****
Mock
EBVzko
KSHV + EBVzko
EBVwt
KSHV + EBVwt
NSG-A2
NSG
E
****
****
****
***
****
Mock
KSHV
EBVzko
KSHV + EBVzko
EBVwt
KSHV + EBVwt
percentage of mice with tumors (%)
100
80
60
40
20
0
1 tumor
≥ 2 tumors
no tumor

Supplementary Figure 1. B cell infection with EBV and KSHV and related tumor formation *in vivo*. Histological stainings of tissue sections from EBV KSHV dual-infected, humanized mice. Arrows indicate examples of double-positive cells, scale bar indicates 50 μm. **A)** EBV EBNA2 (nuclear, brown) and CD20 (surface, red) co-staining in liver (left) and spleen (right) sections. **B)** KSHV LANA (nuclear, brown) and CD20 (surface, red) co-staining in liver (left) and spleen (right) sections. **C)** EBV LMP1 (surface, brown) and KSHV LANA (nuclear, red) co-staining in spleen sections. **D-E)** Lymphoma formation observed at the end point of experiment in organs and peritoneum. **D)** Number of total tumors per mouse. Box plot hinges correspond to 25th and 75th percentiles, shown are median (IQR) and Tukey-style whiskers. **E)** Percentage of mice per group with no, one or two and more tumors. One-way Anova with Tukey correction for multiple comparison. Composite Data from 13 independent experiments with N=17-44 mice per group. ***:p<0.001; ****: p<0.0001 Exact p values from left to right: <0.0001, <0.0001, <0.0001, 0.0006, <0.0001.

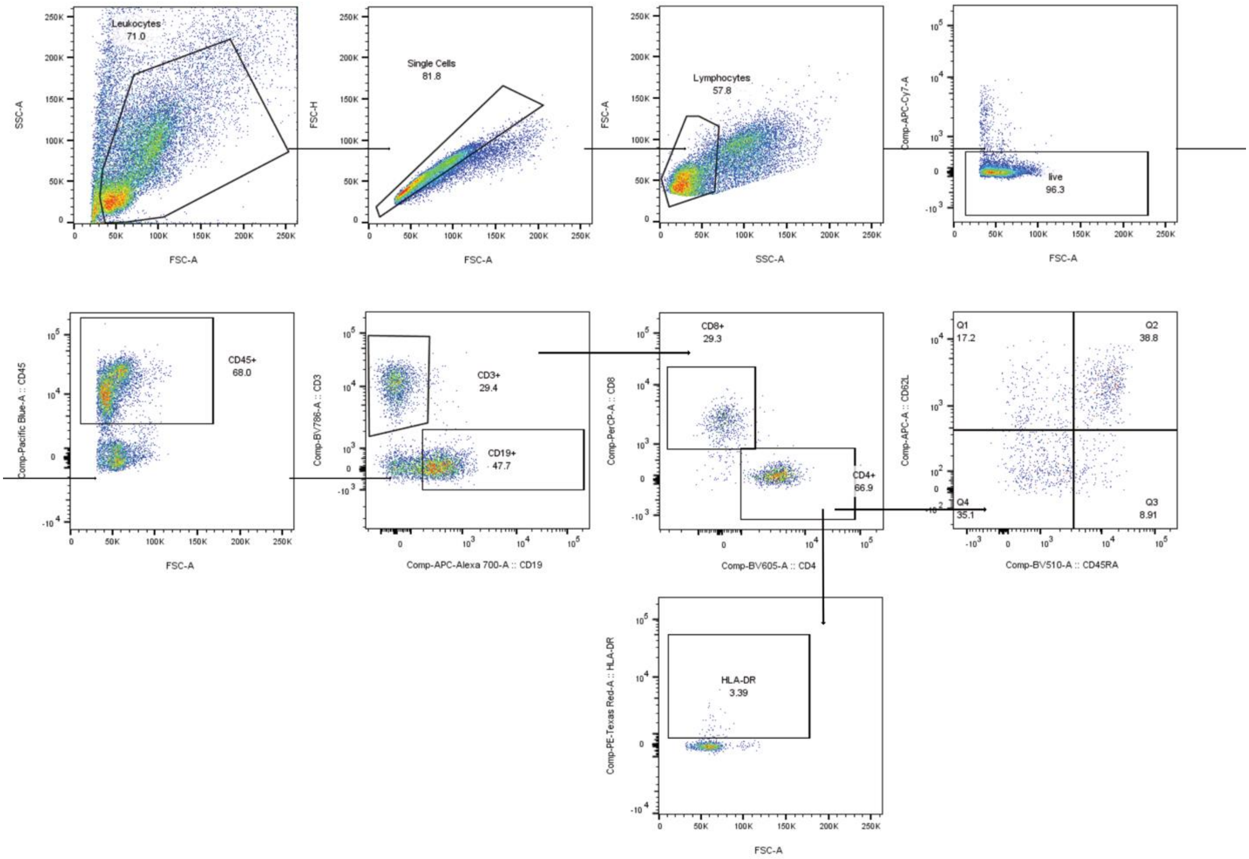

Supplementary Figure 2. Gating strategy for human B and T cells. Single, live lymphocytes were identified by size, granularity and viability dye. Human CD45 was used to gate on human lymphocytes, followed by $CD19^+$ B cells and $CD3^+$ T cells, and the discrimination of $CD3^+$ $CD4^+$ and $CD3^+$ $CD8^+$ T cells. HLA-DR was used to identify activated T cells and CD62L and CD45RA for T cell subsets ($CD62L^+$ $CD45RA^+$: naïve; $CD62L^+CD45RA^-$: central memory; $CD62L^-$ $CD45RA^-$: effector memory; $CD62L^-$ $CD45RA^+$: Temra).

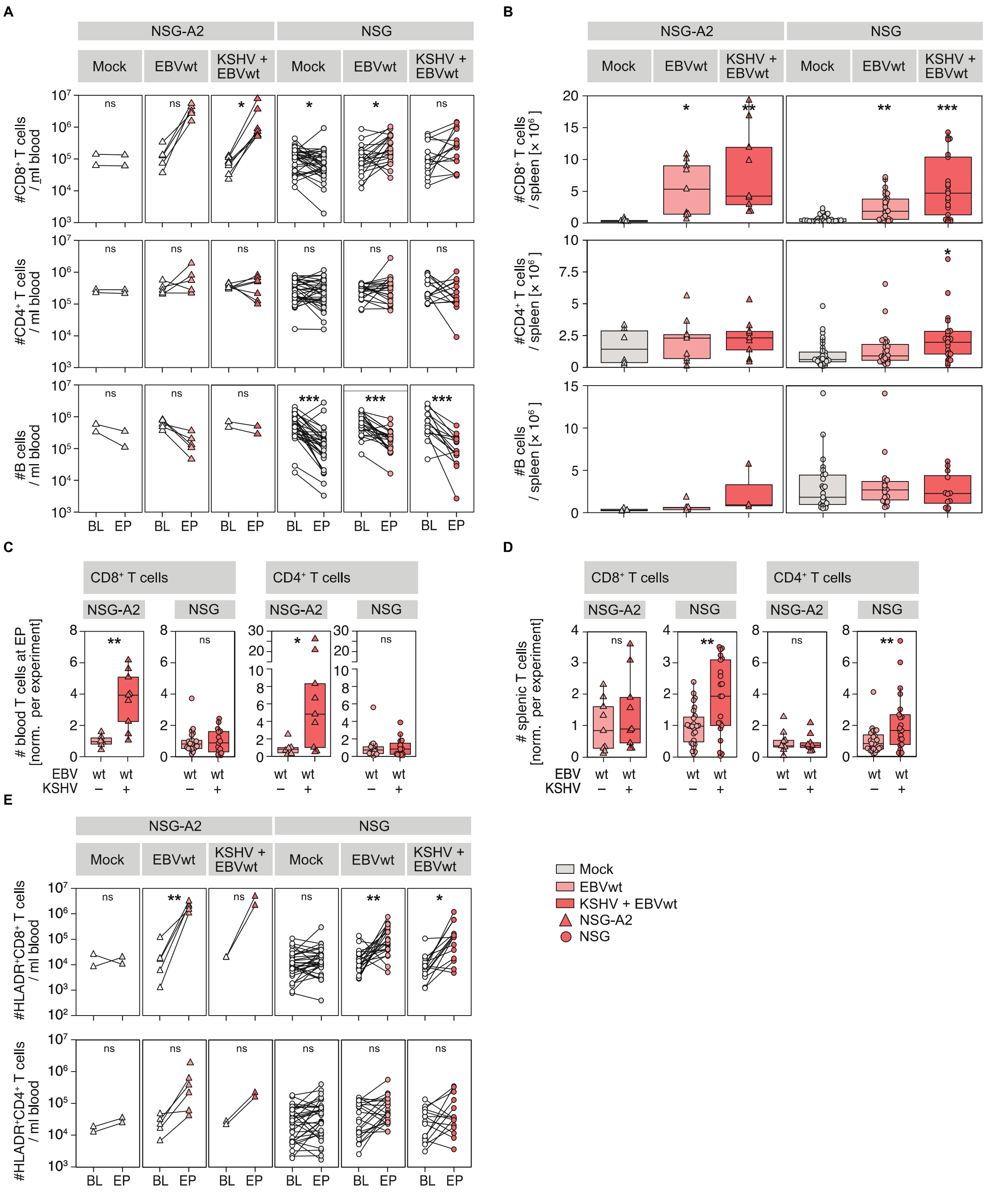

Supplementary Figure 3. T cell and B cell numbers in humanized NSG-A2 and NSG mice. A) Number of human $CD45^{+}CD3^{+}CD8^{+}$, $CD45^{+}CD3^{+}CD4^{+}$ and $CD45^{+}CD19^{+}$ cells/ml blood measured before infection (baseline, BL) and at experiment termination (end point, EP). Composite data from 2 NSG-A2 and 11 (for B cells) or 12 (for T cells) independent experiments in NSG mice, N=2 (NSG-A2 mock), N=5 (NSG-A2 EBVwt), N=2 (B cells) and 7 (T cells) (NSG-A2 KSHV+EBVwt), N= 33 (T cells) or 31 (B cells) (NSG mock) , N=21 (T cells) or 23 (Bcells) (NSG EBVwt) and N=15 (NSG KSHV+EBVwt) mice per group. Two-sided Wilcoxon test. Significant p values from left to right: CD8: 0.01563, 0.04434, 0.01013, 0.0006938; B cells: 1.723e-5, 0.0001335, 3.052e-5.

(Figure legend continues on the next page.)

Supplementary Figure 3. T cell and B cell numbers in A2 and NSG mice. B) Number of human CD45$^+$CD3$^+$CD8$^+$, CD45$^+$CD3$^+$CD4$^+$ and CD45$^+$CD19$^+$ cells per spleen. Composite data from 2 NSG-A2 and 8 (for B cells) and 12 (for T cells) independent experiments in NSG mice, NSG-A2: mock with N=6 (CD8), N=6 (CD4) and N=3 (B cells); EBVwt with N=9 (CD8, CD4), N=5 (B cells); KSHV+EBVwt with N=9 (CD8, CD4), N=3 (B cells); and NSG: mock with N=33 (CD8), N=32 (CD4), N=25 (B cells); EBVwt with N=27 (CD8, CD4), N=19 (B cells) mice per group. Two sided Kruskal Wallis followed by Dunn's test with BF-corrected p-values. Significant p values left to right: CD8: 0.01827, 0.0010399, 1.3251e-3, 9.1292e-7; CD4: 0.01807; **C)** CD45$^+$CD3$^+$CD8$^+$ and CD45$^+$CD3$^+$CD4$^+$ cell numbers measured at 4 weeks p.i. in the blood or **D)** spleen, normalized to the EBVwt single-infected group per experiment. Composite data from **C)** 2 NSG-A2 and 6 NSG independent experiments with N=6 (NSG-A2 EBVwt), N=9 (NSG-A2 KSHV+EBVwt), N=21 (NSG EBVwt), N=15 (NSG KSHV+EBVwt) mice per group, Two sided Mann Whitney U test (MWU), NSG-A2 significant p values are 0.002797 (CD8$^+$) and 0.03596 (CD4$^+$) and **D)** 2 NSG-A2 and 9 NSG independent experiments with N=9 (NSG-A2 EBVwt), N=9 (NSG-A2 KSHV+EBVwt), N=27 (NSG EBVwt), N=21 (NSG, KSHV+EBVwt) mice per group. Two sided MWU, NSG significant p values are 0.006966 (CD8$^+$) and 0.01682 (CD4$^+$). **B-D)** Box plot hinges show 25th and 75th percentile, shown are median (IQR) and Tukey-style whiskers. **E)** Number of human CD45$^+$CD3$^+$CD4$^+$HLA-DR$^+$ and CD45$^+$CD3$^+$CD8$^+$HLA-DR$^+$ cells/ml blood at BL and at experimental EP. Composite data from 2 NSG-A2 and 10 NSG independent experiments with NSG-A2: N=2 (mock), N=5 (EBVwt), N=2 (KSHV+EBVwt) and NSG: N=33 (mock), N=21 (EBVwt), N=15 (KSHV+EBVwt) mice per group. Two sided Wilcoxon test, significant p values left to right for CD45$^+$CD3$^+$CD8$^+$HLA-DR$^+$: 0.005593, 0.004788, 0.02879. *:p<0.05; **:p<0.01; ***:p>0.001.

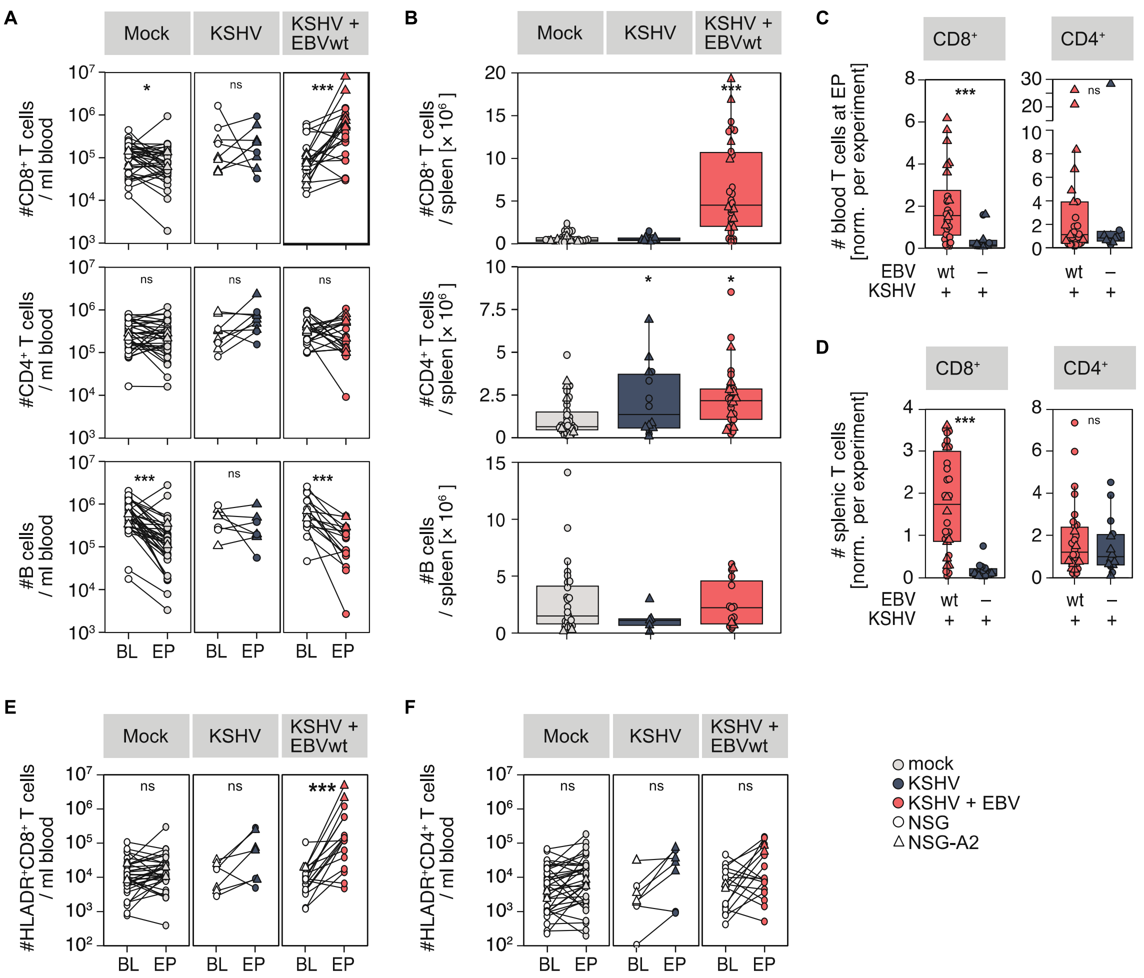

Supplementary Figure 4. T cell and B cell numbers for KSHV single-infected mice. A) Number of human $CD45^+CD3^+CD8^+$, $CD45^+CD3^+CD4^+$ and $CD45^+CD19^+$ cells / ml blood measured before infection (baseline, BL) and at experiment termination (end point, EP). Mock: N=35 (T cells) and N=36 (B cells), KSHV: N=9 (T cells) and N=7 (B cells), KSHV+EBVwt: N=22 (T cells) and N=16 (B cells) mice per group. Two sided Wilcoxon Test, significant p values left to right: $CD8^+$: 0.04383; 5.53e-5. B cells: 6.657e-7, 3.052e-3. **B)** Number of human $CD45^+CD3^+CD8^+$, $CD45^+CD3^+CD4^+$ and $CD45^+CD19^+$ cells per spleen. Mock: N=38 (T cells) and N=28 (B cells), KSHV: N=14 (T cells) and N=5 (B cells), KSHV+EBVwt: N=30 (T cells) and N=15 (Bcells) mice per group. Two sided Kruskal Wallis followed by Dunn's test with BF-corrected p-values. Significant p values left to right: $CD8^+$: 6.577e-6, $CD4^+$: 0.03569, 0.006. **C)** $CD45^+CD3^+CD8^+$ and $CD45^+CD3^+CD4^+$ cell numbers measured at 4 weeks p.i. in the blood with N=24 (KSHV+EBVwt) and N=10 (KSHV $CD8^+$)/9 (KSHV $CD4^+$) mice per group or **D)** in spleen with N=30 (KSHV+EBVwt) and N=14 (KSHV) mice per group, normalized to the EBVwt single-infected group per experiment. Box plot hinges represent 25th and 75th percentile, shown are median (IQR) and Tukey-style whiskers. Two sided MWU. Significant p values are **C)** 2.992e-7 and **D)** 0.002007. **E)** Number of human $CD45^+CD3^+CD4^+HLA\text{-}DR^+$ and **F)** $CD45^+CD3^+CD8^+HLA\text{-}DR^+$ cells / ml blood at BL and at experimental EP. N=35 (mock), N=7 (KSHV) and N=17 (KSHV+EBVwt) mice per group. Two sided Wilcoxon test. Significant p value is 2.889e-5. **A-F)** Composite data from 13 independent experiments. *:p<0.05; **:p<0.01; ***:p>0.001.

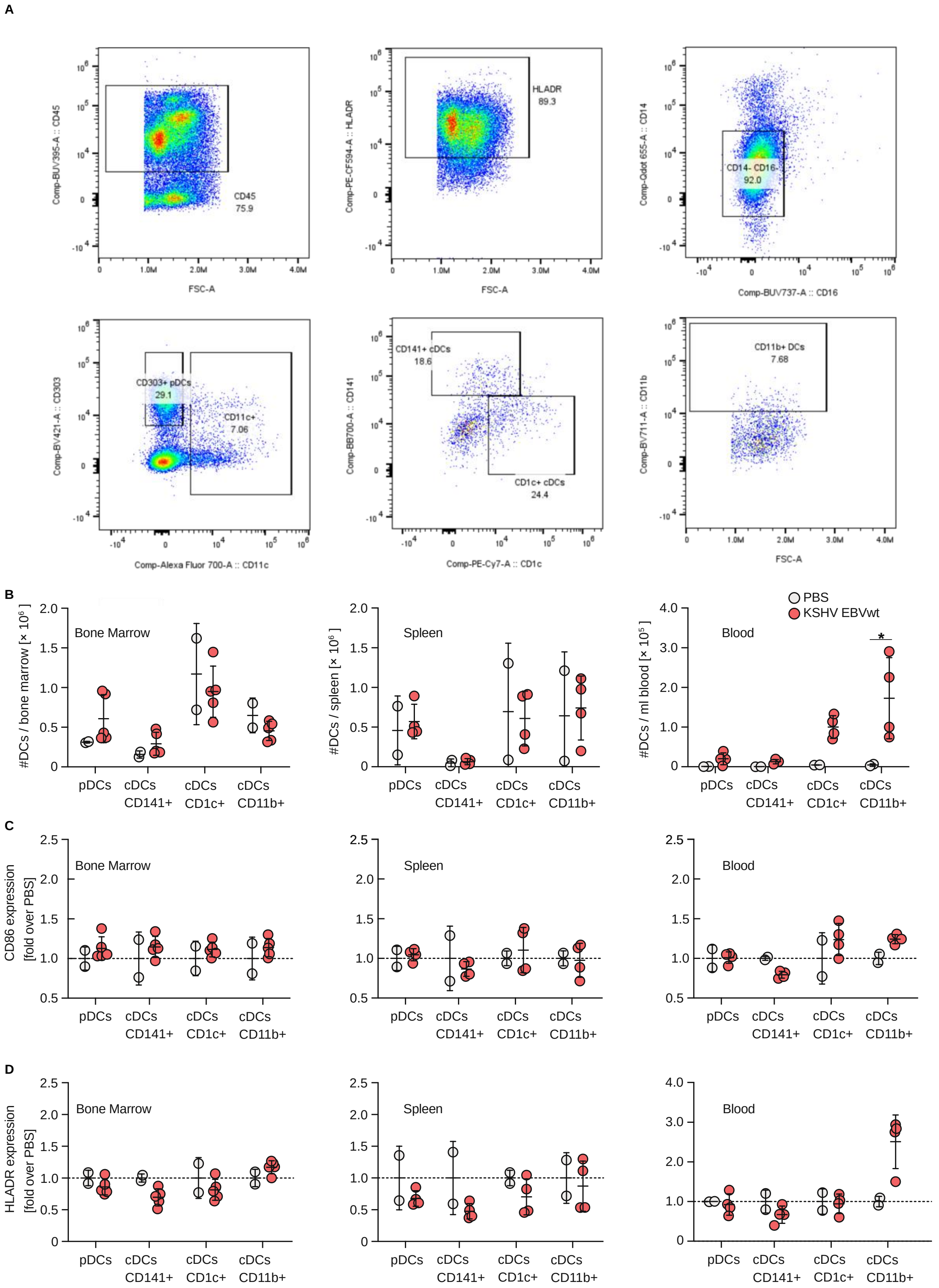

Supplementary Figure 5. Dendritic cells in humanized mice infected with EBV and KSHV.

(Full Figure Legend on the next page.)

Supplementary Figure 5. Dendritic cells in humanized mice infected with EBV and KSHV. **A)** Gating strategy for human DCs. Single live leukocytes were gated for huCD45$^+$ cells. From there, HLA-DR$^+$ cells were gated and CD14$^-$CD16$^-$ cells were divided into CD303$^+$CD11c$^-$ pDCs and CD11c$^+$ DCs. CD11c$^+$ DCs were further separated into CD141$^+$ cDCs, CD1c$^+$ cDCs and CD11b$^+$ cDCs. **B)** DC subsets (pDC, CD141$^+$ cDCs, CD1c$^+$ cDCs and CD11b$^+$ cDCs) for bone marrow, spleen and blood are shown per organ (bone marrow, spleen) or ml. two sided MWU, significant p value is 0.02866. **C)** displays CD86 expression as fold over PBS for the different DC subsets and organs. **D)** displays HLA-DR expression as fold over PBS for the different DC subsets and organs. N=2 (PBS) and N=5 (bone marrow) /4 (Spleen, blood) (KSHV+EBVwt) animals from 1 experiment.

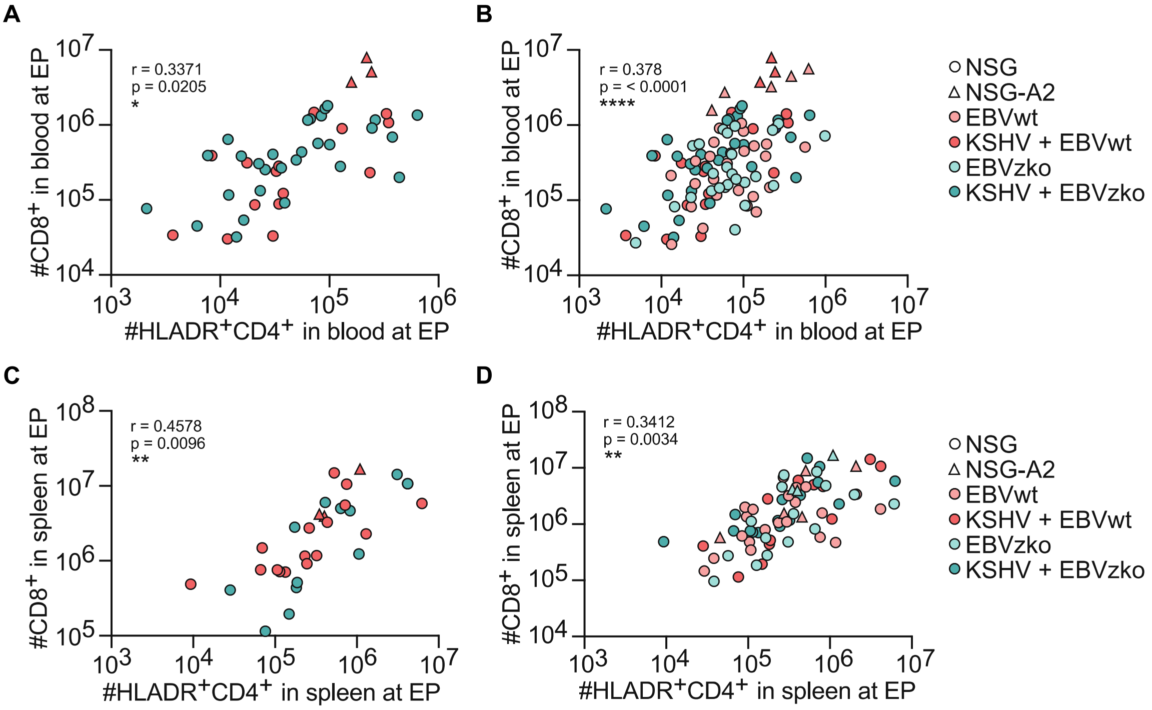

Supplementary Figure 6. CD4⁺ T cell activation correlates with CD8⁺ T cell expansion. A)-B) Number of activated (HLA-DR⁺) CD4⁺ T cells in blood at end point correlates with the number of blood CD8⁺ T cells at endpoint for both **A)** KSHV/EBV dual-infected mice, and **B)** EBV single-infected and KSHV/EBV dual-infected mice. Pooled data from **A)** 12 and **B)** 13 independent experiments with N=47 and N=104 mice, respectively. **C)-D)** Number of activated (HLA-DR⁺) CD4⁺ T cells correlates with the number of CD8⁺ T cells in spleen for **C)** KSHV/EBV dual-infected mice and **D)** EBV single-infected and KSHV/EBV dual-infected mice. Pooled data from 9 independent experiments, N=31 and N=72 mice, respectively. Pearson r coefficient and two-sided p value test; ns: p>0.05; *: p<0.05; **: p<0.01; ***:p<0.001; ****: p<0.0001.

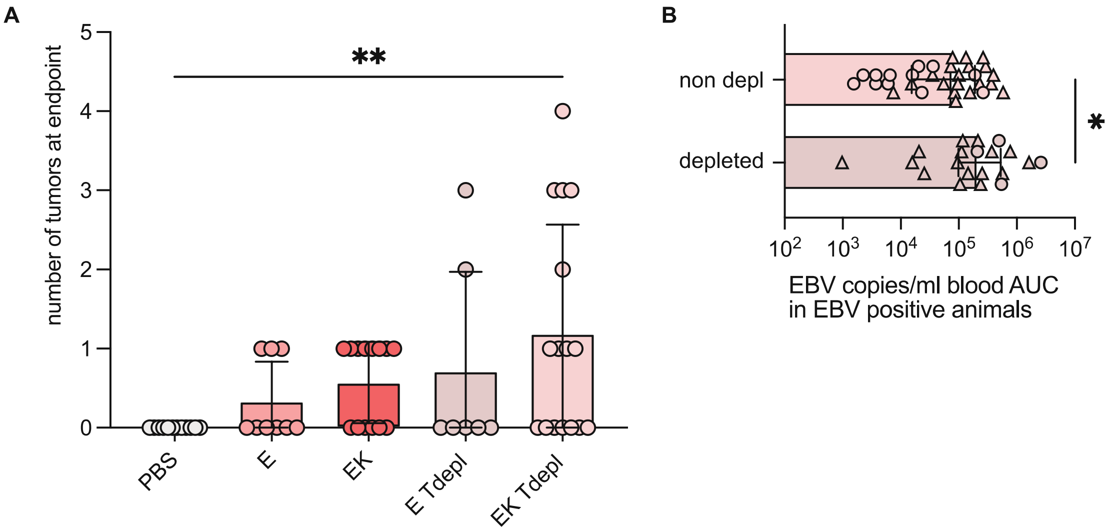

Supplementary Figure 7. T cell depletion increases EBV viral loads. A) Tumor formation from 3 independent experiments with N=13 Mock- infected, N=9 EBV- infected, N=14 KSHV/EBV co-infected, N=7 EBV- infected T cell depleted and N=15 KSHV/EBV co-infected T cell depleted animals. One-way Anova, significant p value is 0.0096. **B)** EBV copies/ml blood in EBV positive (EBV and KSHV/EBV) mice with or without T cell depletion over the course of the experiment depicted as AUC, two sided MWU, significant p value is 0.0129. Pooled data from 3 independent experiments with N=20 (depleted) and N=31 (non depl) mice per group.

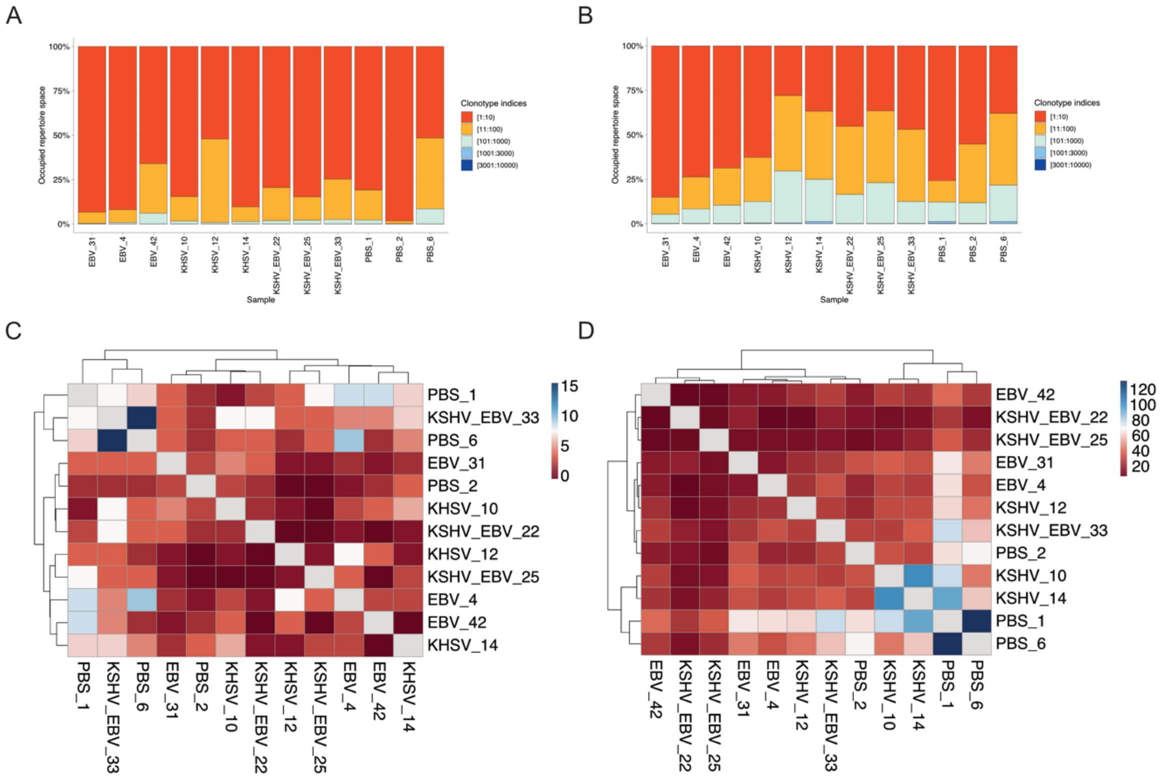

Supplementary Figure 8. TCR sequence analysis for CD8^{+} and CD4^{+} T cells from humanized mice. CD8^{+} and CD4^{+} T cells were sorted using the BD Aria 5L sorter from spleens of huNSG-A2 mice infected with Mock, EBVzko, KSHV or KSHV/EBVzko and TCR sequencing was performed. Top clonal composition of **A)** CD8^{+} and **B)** CD4^{+} cells displayed via occupied repertoire space of the top 1-10, top 11-100, top 101-1000 and top 1001-3000 and top 3001-10000 clones. Repertoire overlap of **C)** CD8^{+} and **D)** CD4^{+} T cell clones showed that some clones overlap amongst animals in each group, but mostly non-abundant clones were observed. Mice come from one experiment, TCR sequencing was performed for 3 mice per group.

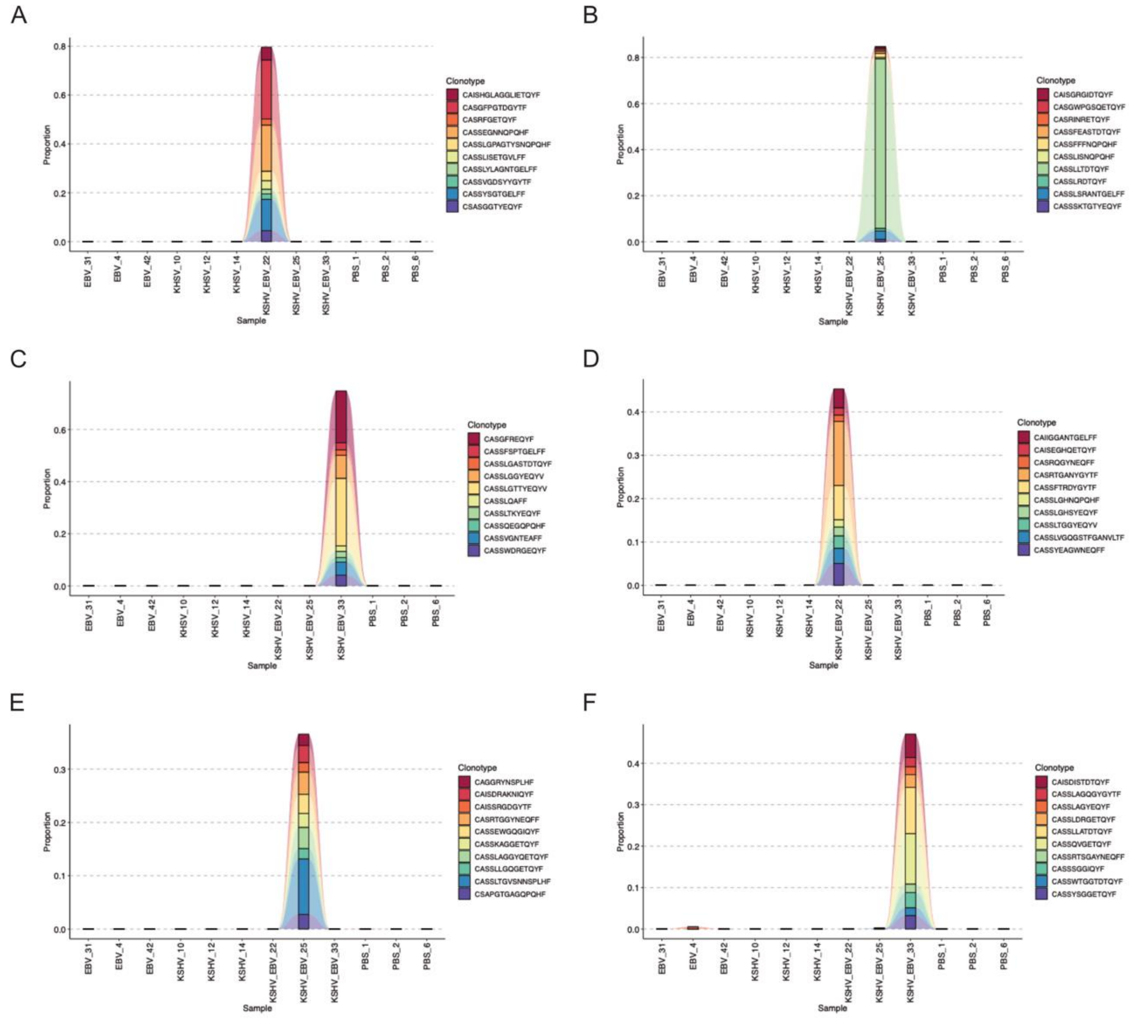

Supplementary Figure 9. TCR sequence overlap analysis for CD8^{+} and CD4^{+} T cells from humanized mice. CD8^{+} and CD4^{+} T cells were sorted using the BD Aria 5L sorter from spleens of huNSG-A2 mice infected with Mock, EBVzko, KSHV or EBVzko+KSHV and TCR sequencing was performed. Overlap analysis for **A-C)** the top 10 CD8^{+} TCRs and **D-F)** the top 10 CD4^{+} TCRs of EBVzko+KSHV mice only showed one CD4^{+} clone that was present in the Top 10 TCRs of Mouse KSHV_EBV_33 and EBV_4. Mice come from one experiment, TCR sequencing was performed for 3 mice per group.

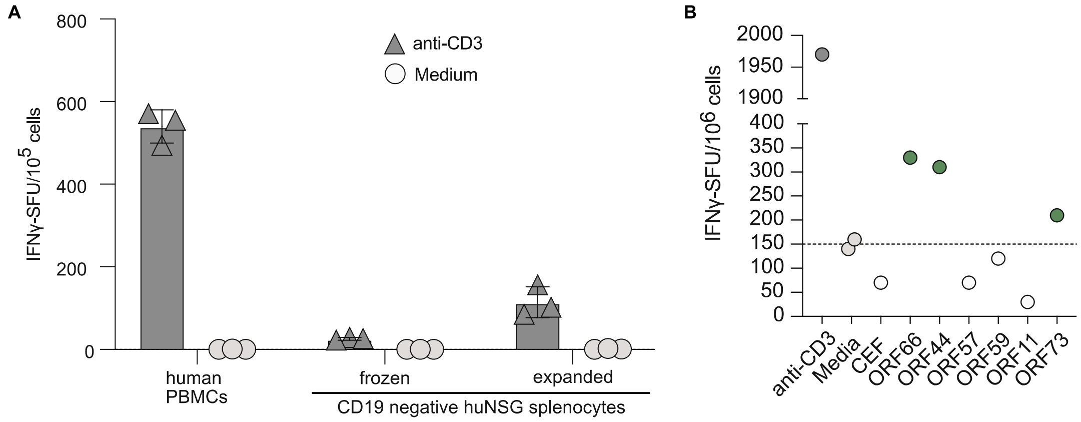

Supplementary Figure 10. T cell specificity to KSHV peptide pools in mice dual-infected with KSHV and EBV. A) IFNγ ELISpot results of CD19-negative splenocytes from KSHV/EBVzko infected humanized mice after anti-CD3 or medium stimulation overnight directly after thawing or after expansion of thawed cells. Reactivity was not retained directly after thawing but could be recovered upon expansion. **B)** IFNγ ELISpot results of CD19-negative splenocytes after stimulation with KSHV peptide pools over night after expansion with CD3/CD28 beads.

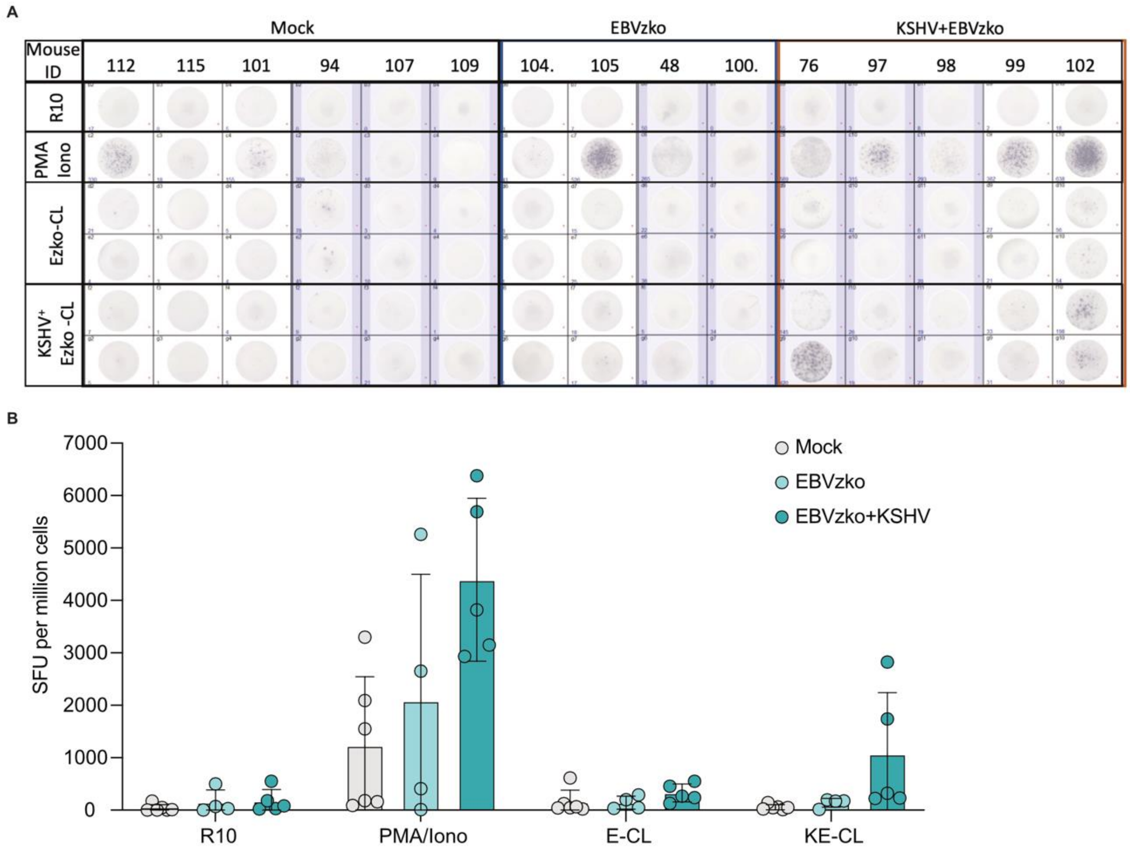

Supplementary Figure 11. T cell responses against KE-CL and E-CL in individual infected humanized mice. A) IFNγ ELISpot results of CD19-negative splenocytes from Mock, EBVzko or KSHV/EBVzko infected humanized mice upon overnight co-culture with R10, PMA/Iono, E-CL or KE-CL. Depicted are mice from one donor, values shown for E-CL and KE-CL are mean values from technical replicates. N=6 (mock), N=4 (EBVzko) and N=5 (KSHV+EBVzko) mice per group. **B)** Quantification of the ELISpot with spot forming units counted by the software. Shown are individual values for R10 and PMA/Iono and means of technical replicates for E-CL and KE-CL stimulation. Error bars represent standard deviation.

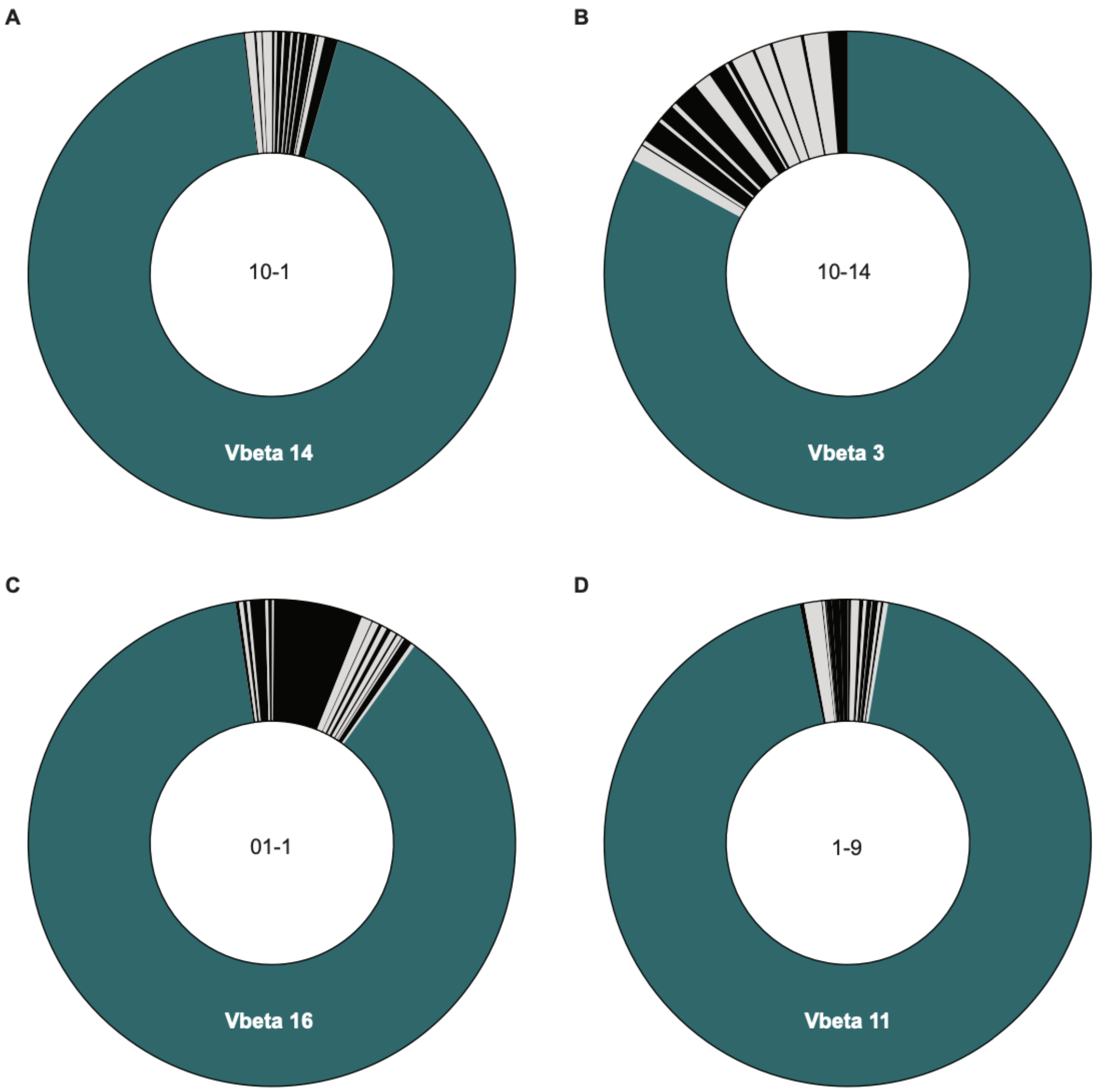

Supplementary Figure 12. T cell subpopulation Vβ staining Pie charts of cells that stained positive for the indicated TCR Vβ sequences in flow cytometry. Percentage is based on all cells of the parent population ($CD8^+$ or $CD4^+$ T cells) that showed any staining of TCR Vβ sequences. Shown is the TCR Vβ analysis for **A)** $CD4^+$ subpopulation 10-1, **B)** $CD4^+$ subpopulation 10-14, **C)** $CD8^+$ subpopulation 01-1 and **D)** $CD8^+$ subpopulation 1-9.

A

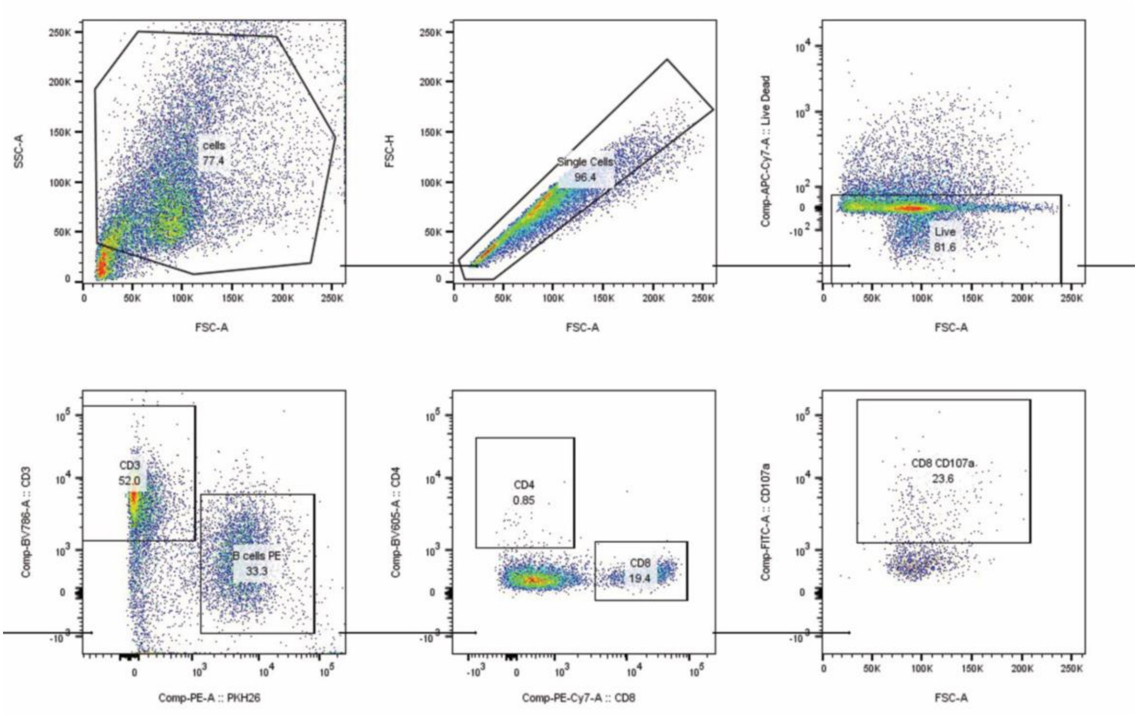

B

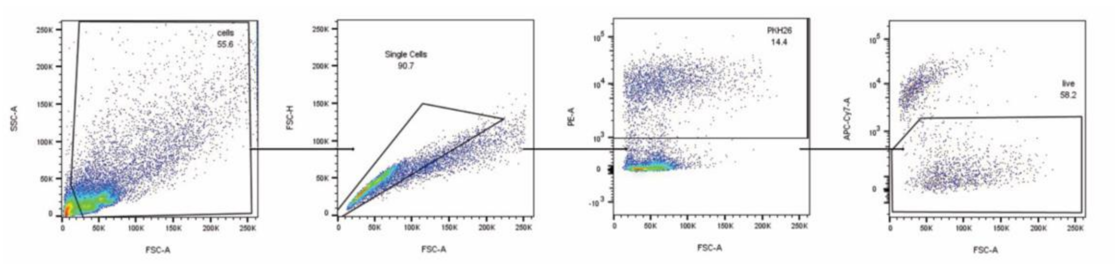

Supplementary Figure 13. Gating strategy for T cell degranulation and T cell killing assay. A) CD107a was used to assess degranulation of live $CD3^{+}CD8^{+}$ and $CD3^{+}CD4^{+}$ T cells upon coculture with B cells (stained with PKH-27 dye). **B)** Target cells were identified using PKH-26 stain (PE) and frequency of the live cells was assessed by viability stain.

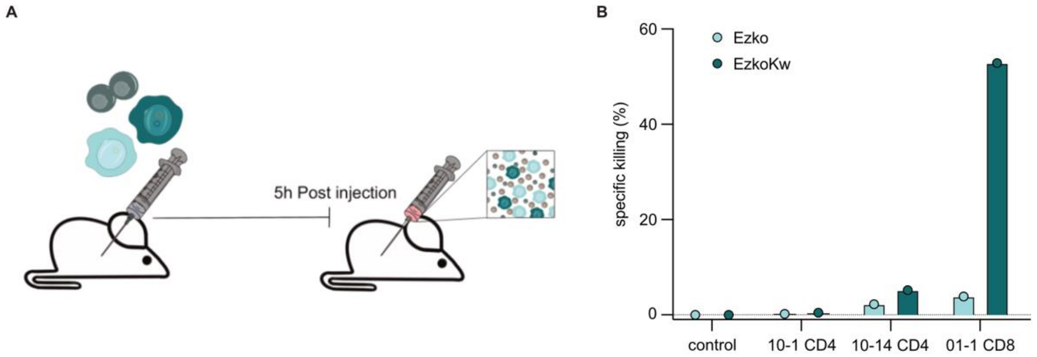

Supplementary Figure 14. CD8⁺ T cell subpopulation 01-1 specifically kills KE-CL *in vivo*. A) Schematic of the *in vivo* killing assay, where T cells along with labeled E-CL and KE-CL were injected i.p. into NSG mice and collected after 5h by peritoneal lavage. **B)** Specific killing of the extracted cell populations was calculated using the percentage of dead cells amongst the pre-labeled E-CL and KE-CL compared to control (no T cells). 1 experiment with N=1 mouse per group.

Supplementary Table S1. Percentage of positive T cells from the Vβ staining (Wei nomenclature). Given are the percentages of the $CD4^+$ or $CD8^+$ T cells that were positive for the stained Vβ.

**TCR Vβ**	**10-1 ($CD4^+$)**	**10-14 ($CD4^+$)**	**01-1 ($CD8^+$)**	**1-9 ($CD8^+$)**
**Vβ 3**	0.193	82.843	0.142	0.033
**Vβ 5.1**	0.157	1.069	5.898	0.183
**Vβ 1**	0.350	0.000	0.700	0.538
**Vβ 4**	0.145	0.107	0.078	0.274
**Vβ 2**	0.420	0.285	0.430	0.213
**Vβ 5.3**	0.133	1.550	0.238	0.325
**Vβ 5.2**	0.326	0.223	0.350	0.000
**Vβ 7**	0.111	1.078	0.318	0.000
**Vβ 6.7**	0.350	0.312	0.370	0.049
**Vβ 9**	0.098	1.755	0.140	0.386
**Vβ 8**	0.600	0.000	0.300	0.193
**Vβ 13.1**	0.107	1.158	0.079	0.162
**Vβ 13.6**	0.121	1.203	0.127	0.304
**Vβ 11**	0.386	0.196	0.556	94.157
**Vβ 12**	0.881	0.338	0.254	0.304
**Vβ 16**	0.028	1.452	87.622	1.147
**Vβ 14**	93.718	0.205	0.238	0.071
**Vβ 18**	0.120	1.015	0.270	0.152
**Vβ 17**	0.590	0.205	0.238	0.099
**Vβ 21.3**	0.157	1.888	0.207	0.000
**Vβ 20**	0.326	0.232	1.017	0.355
**Vβ 23**	0.091	1.559	0.191	0.528
**Vβ 22**	0.592	1.327	0.238	0.528

Supplementary Table S2. Spot forming units of the IFNγ ELISpot for all KSHV peptide pools. Given are the spot forming units from the IFNγ ELISpot of the T cells from the IFNγ capture assay (150 000 cells per well) after co-culture with KSHV peptide pools.

Peptide pool	replicate 1	replicate 2	replicate 3	Peptide pool	replicate 1	Peptide pool	replicate 1
anti-CD3	18	29		ORF17	0	ORF64-3	0
CEF	1	1		ORF20	0	ORF68	0
EBV	1	1		ORF23	0	K12	0
SIV-CM9	1	1		ORF26	0	K13	0
Medium	0	0	0	ORF28	0	ORF74	0
ORF70	1			ORF29b	0	K15	0
K6	20			ORF31	0	K1	0
ORF27	1			ORF32	0	K5	0
ORF31	1			ORF33	0	ORF16	0
ORF36	1			ORF29a	0	ORF18	0
ORF41	1			ORF34	0	ORF30	0
ORF64-1	2			ORF39	0	ORF35	0
ORG66	2			ORF40	0	ORF42	0
K14	1			ORF37	0	ORF46	0
ORF38	1			ORF56	0	ORF47	0
K8	1			ORF43	0	ORF55	0
ORF52	2			ORF45	0	ORF57	0
ORF63	1			ORF48	0	ORF67	0
ORF65	1			ORF49	0	ORF72	0
ORF69	1			ORF50	0	ORF6	0
ORF25	1			ORF53	0	ORF8	0
ORF73	1			ORF54	0	ORF21	0
K8.1	0			K9	0	ORF22	0
ORF4	0			K10	0	ORF24	0
ORF7	0			K11	0	ORF44	0
ORF16	0			ORF58	0	ORF41	0
ORF11	0			ORF59	0	ORF75	0
K2	0			ORF60	0	ORF9	0
ORF2	0			ORF62	0	K7	0
K3	0			ORF64-2	0	ORF19	0
K4	0						

Supplementary Table S3. Key Resources Table. All reagents and 204 materials used to generate the data of this manuscript.

REAGENT or RESOURCE	SOURCE	IDENTIFIER
**Antibodies and dilutions**		
BUV395 mouse anti-human CD45 (Clone: HI30) 1:100	BD Bioscience	Cat#563792; RRID:AB_2744400
BUV737 mouse anti-human CD16 (Clone: 3G8) 1:100	BD Bioscience	Cat#612786 RRID:AB_2833077
PB mouse anti-human CD45 (Clone: HI30) 1:500	BioLegend	Cat#304029; RRID:AB_2174123
BB700 mouse anti-human CD141 (Clone: 1A4) 1:50	BD Biosciences	Cat# 742245 RRID:AB_2740668
BV421 mouse anti-human CD303 (Clone: V24-785) 1:100	BD Biosciences	Cat# 566428 RRID:AB_2744264
BV510 mouse anti-human CD45RA (Clone: HI100) 1:100	Biolegend	Cat# 304141 RRID: AB_ 2561384
BV510 mouse anti-human CD86 (Clone: IT2.2) 1:100	Biolegend	Cat# 305431 RRID:AB_2562064
BV570 mouse anti-human CD4 (Clone: RPA-T4) 1:100	Biolegend	Cat# 300533, RRID:AB_10896788
BV605 mouse anti-human CD3 (Clone: Okt.3) 1:100	Biolegend	Cat# 317322, RRID:AB_2561911
BV605 mouse anti-human CD4 (Clone: OKT4) 1:100	Biolegend	Cat#317438; RRID:AB_11218995
BV650 mouse anti-human CD8 (Clone: SK1) 1:100	Biolegend	Cat# 344729 RRID: AB_2564509
BV711 mouse anti-human CD11b (Clone: ICRF44) 1:100	Biolegend	Cat# 301343 RRID:AB_2563792
BV785 mouse anti-human CD3 (Clone: OKT3) 1:100	Biolegend	Cat#317330; RRID:AB_2563507
FITC mouse anti-human HLADR (Clone: L243) 1:200	Biolegend	Cat# 307604 RRID:AB_ 314682
FITC mouse anti-human CD107a (Clone: H4A3) 1:50	BD Bioscience	Cat#555800; RRID:AB_396134
Per CP mouse anti-human CD8 (Clone: SK1) 1:100	Biolegend	Cat#344708; RRID:AB_1967149
PE mouse anti-human CD3 (Clone: UCHT1) 1:100	Biolegend	Cat# 980008 RRID:AB_ 2810818
PE mouse anti-human CD64 (Clone: 10.1) 1:100	Biolegend	Cat# 305007 RRID:AB_314491
PE CF594 mouse anti-human HLADR (Clone: G46-6) 1:250	BD Biosciences	Cat# 562304 RRID:AB_11154415
PE/TexasRed mouse anti-human CD19 (Clone: SJ25-C1) 1:50	ThermoFisher Scientific	Cat# MHCD1917 RRID:AB_ 10372040
PE-Cy7 mouse anti-human CD19 (Clone: HIB19) 1:50	Biolegend	Cat# 302216 RRID:AB_ 314246
PE-Cy7 mouse anti-human HLADR (Clone: L243) 1:200	Biolegend	Cat# 307615 RRID: AB_493589
PE-Cy7 mouse anti-human CD62L (Clone: DREG-56) 1:100	Biolegend	Cat# 304821 RRID:AB_ 830800
PE-Cy7 mouse anti-human CD1c (Clone: L161) 1:50	Biolegend	Cat# 331516 RRID:AB_2275574
APC mouse anti-human CD62L (Clone: DREG-56) 1:100	BD Bioscience	Cat# 304809 RRID:AB_314469
Alexa Fluor 700 mouse anti-human CD19 (Clone: HIB19) 1:50	Biolegend	Cat#302226; RRID:AB_493751

Alexa Fluor 700 mouse anti-human CD11c (Clone: B-ly6) 1:50	BD Biosciences	Cat# 561352 RRID:AB_10612006
APC-Cy7 mouse anti-human CD4 (Clone: RPA-T4) 1:100	Biolegend	Cat# 300517 RRID:AB_314085
QDot 655 mouse anti-human CD14 (Clone: TuK4) 1:100	Thermo Fisher Scientific	Cat#Q10056 RRID:AB_2556446
InVivoMAb anti-human CD4 (Clone: OKT4) depletion antibody	Bio X Cell	Cat# BE0003-2, RRID:AB_1107638
InVivoMAb anti-human CD8 (Clone: OKT8) depletion antibody	Bio X Cell	Cat# BE0004-2, RRID:AB_1107673
PE rat anti-human Vβ1 (Clone: BL37.2) 1:30	Beckman coulter	Cat# IM2355 RRID:AB_131329
PE mouse anti-human Vβ2 (Clone: MPD2D5) 1:30	Beckman coulter	Cat# IM2213 RRID:AB_131311
FITC mouse anti-human Vβ3 (Clone: CH92) 1:30	Beckman coulter	Cat# IM2372 RRID:AB_131046
PE rat anti-human Vβ4 (Clone: WJF24) 1:30	Beckman coulter	Cat# IM3602 RRID:AB_131344
FITC mouse anti-human Vβ5.1 (Clone: IMMU 157) 1:30	Beckman coulter	Cat# IM1552 RRID:AB_131023
FITC mouse anti-human Vβ5.2 (Clone: 36213) 1:30	Beckman coulter	Cat# IM2286 RRID:AB_131322
PE mouse anti-human Vβ5.3 (Clone: 3D11) 1:30	Beckman coulter	Cat# IM2002 RRID:AB_131230
PE mouse anti-human Vβ7.1 (Clone: ZOE) 1:30	Beckman coulter	Cat# IM2287 RRID:AB_131323
FITC mouse anti-human Vβ8 (Clone: 56C5.2) 1:30	Beckman coulter	Cat# IM1233 RRID:AB_130922
PE mouse anti-human Vβ9 (Clone: FIN9) 1:30	Beckman coulter	Cat# IM2003 RRID:AB_131193
FITC mouse anti-human Vβ11 (Clone: C21) 1:30	Beckman coulter	Cat# IM1586 RRID:AB_131027
PE mouse anti-human Vβ12 (Clone: VER2.32.1) 1:30	Beckman coulter	Cat# IM2291 RRID:AB_131198
PE mouse anti-human Vβ13.1 (Clone: IMMU 222) 1:30	Beckman coulter	Cat# IM2292 RRID:AB_131326
FITC mouse anti-human Vβ13.6 (Clone: JU74.3) 1:30	Beckman coulter	Cat# IM1330 RRID:AB_131012
PE mouse anti-human Vβ14 (Clone: CAS1.1.3) 1:30	Beckman coulter	Cat# IM2047 RRID:AB_131304
FITC mouse anti-human Vβ16 (Clone: TAMAYA1.2) 1:30	Beckman coulter	Cat# IM1560 RRID:AB_130875
FITC mouse anti-human Vβ17 (Clone: E17.5F3.15.13) 1:30	Beckman coulter	Cat# IM1234 RRID:AB_131007
PE mouse anti-human Vβ18 (Clone: BA62.6) 1:30	Beckman coulter	Cat# IM2049 RRID:AB_131305
PE mouse anti-human Vβ20 (Clone: ELL1.4) 1:30	Beckman coulter	Cat# IM2295 RRID:AB_131328
FITC mouse anti-human Vβ21.3 (Clone: IG125) 1:30	Beckman coulter	Cat# IM1483 RRID:AB_131021
FITC mouse anti-human Vβ22 (Clone: IMMU 546) 1:30	Beckman coulter	Cat# IM1484 RRID:AB_131022
PE mouse anti-human Vβ23 (Clone: AF23) 1:30	Beckman coulter	Cat# IM2004 RRID:AB_131302
Goat anti-human IgM-UNLB (polyclonal)	Southern Biotech	Cat# 2020-01 RRID:AB_2795599
Mouse anti-EBNA2 (Clone: PE2)	Abcam	Cat#ab90543 RRID: AB_2049594

Rat anti-LANA (Clone: LN53)	Clinisciences	Cat#Mob395; RRID:AB_2860565
Rabbit anti-human CD20 (Clone: SP32)	Cell Marque	Cat#120R-16 RRID:AB_2860563
Mouse anti-human IRF4/MUM1 (Clone: MUM1p)	Clinisciences	Cat#Mob420 RRID:AB_2861384
Mouse anti-human CD68 (Clone: 514H12)	Leica Biosystems	Cat# NCL-L-CD68 RRID: AB_563622
Rabbit anti-human CD3 (Clone: SP7)	Diagnostic Biosystems	Cat# RMAB005 RRID:AB_2860564
Mouse anti-EBV LMP1 (Clone: CS1-4)	Abcam	Cat# ab78113 RRID: AB_1566182
**Bacterial and Virus Strains**		
EBV B95-8-GFP (EBVwt) produced in HEK293	(Delecluse et al., 1998)	N/A
EBV B95-8-BZLF1KO-GFP (EBVzko) produced in HEK293	(Feederle et al., 2000)	N/A
rKSHV.219	(Vieira and O'Hearn, 2004)(Kati et al., 2015)	N/A
KSHV BAC16 (rKSHV.219) produced in iSLK	(Brulois et al., 2012)	N/A
**Biological Samples**		
Human Fetal Liver samples	Advanced Bioscience Resources	N/A
Human Blood Buffy Coats	Blutspende Zürich Blutausgabe	N/A
**Chemicals, Peptides, Recombinant Proteins and beads**		
Zombie Aqua Fixable Viability Dye	Biolegend	Cat#423102
Zombie NIR Fixable Viability Dye	Biolegend	Cat#423106
Live Dead blue Vixable Dead cell stain	Invitrogen	Cat#L23105
TPA (12-O-Tetradecanoylphorbol 13-acetate)	Sigma-Aldrich	Cat#P1585
ACK lysis buffer	Thermo Fischer	Cat#A1049201
Ficoll-Paque	GE-Healthcare	Cat#17-1440-02
CD19 microbeads	Miltenyi Biotech	Cat#130-050-301
Recombinant human IL-2	PeproTech	Cat# 200-02
Opal 540 reagent pack	Perkin Elmer	Cat#FP1494001KT
Opal 620 reagent pack	Perkin Elmer	Cat# FP1495001KT
Opal 650 reagent pack	Perkin Elmer	Cat# FP1496001KT
Opal 690 reagent Pack	Perkin Elmer	Cat# FP1497001KT
Dapi	Perkin Elmer	Cat# CS1-0127-2ML
Cell Trace Violet	Invitrogen	Cat# C34557
Cell Trace Far Red	Invitrogen	Cat# C34564
Cell Trace Blue	Invitrogen	Cat# C34568
**Critical Commercial Assays**		
DNeasy Blood & Tissue Kit	Qiagen	Cat#69506
IFNγ ELISpot Assay Flex HRP kit	MabTech	Cat#3420-2H
IFNγ ELISA Assay HRP kit	MabTech	Cat#3420-1H-6
IgG ELISA Assay	Sigma Aldrich	Cat#RAB0001
IgM ELISA Assay	Abcam	Cat#ab137982
IFNγ Secretion Assay - Detection Kit	Miltenyi Biotech	Cat#130-054-202
**Experimental Models: Cell Lines**		
Brk.219	(Kati et al., 2013)	N/A

Raji cells	American Type Culture Collection	CCL-86, cultured in our laboratory
HEK293T	American Type Culture Collection	CRL-3216, cultured in our laboratory
**Experimental Models: Organisms/Strains**		
NOD.Cg-*Prkdc*^{scid} *Il2rg*^{tm1Wjl}/SzJ (NSG mice)	The Jackson Laboratory	Stock#005557
NOD.Cg-*Mcph1*$^{Tg(HLA\text{-}A2.1)1Enge}$ *Prkdc*^{scid} *Il2rg*^{tm1Wjl}/SzJ (NSG-A2 mice)	The Jackson Laboratory	Stock#009617
**Oligonucleotides**		
EBV BamHI forward primer: 5′-CTTCTCAGTCCAGCGCGTTT-3′	modified from (Berger et al., 2001)	N/A
EBV BamHI reverse primer: 5′-CAGTGGTCCCCCTCCCTAGA-3′	modified from (Berger et al., 2001)	N/A
EBV BamHI probe: 5′-(FAM)-CGTAAGCCAGACAGCAGCCAATTGTCAG-(TAMRA)-3′	modified from (Berger et al., 2001)	N/A
KSHV ORF26 forward primer: 5′-GCTCGAATCCAACGGATTTG-3′	modified from (Tedeschi et al., 2001)	N/A
KSHV ORF26 reverse primer: 5′-AATAGCGTGCCCCAGTTGC-3	modified from (Tedeschi et al., 2001)	N/A
KSHV ORF26 probe: 5'-(FAM)-TTCCCCATGGTCGTGCCTC-(BHQ-1)-3'	modified from (Tedeschi et al., 2001)	N/A
**Software and Algorithms**		
R (v3.6.3 and v4.3.1)	R Core Team	www.r-project.org,
RStudio (v1.2.5033 and 2023.06.2+561)	RStudio	https://rstudio.com
FlowJo (v10.6.2)	FlowJo	www.flowjo.com
Inform (v2.4.8)	PerkinElmer	N/A
Phenochart (v1.0)	PerkinElmer	N/A
Vectra 3.0	PerkinElmer	N/A
CFX Manager Software	BioRad	N/A
Prism (v8.4.3, v9.5.0)	GraphPad Software	www.graphpad.com
SPICE - Simplified Presentation of Incredibly Complex Evaluations	(Roederer et al., 2011)	https://niaid.github.io/spice
MiXCR software pipeline, version 3.0.13	(Bolotin et al., 2015)	https://mixcr.comImm
VDJtools version 1.2.1	(Shugay et al., 2015)	https://vdjtools-doc.readthedocs.io/en/master/
Immunarch	(Nazarov, 2020)	https://immunarch.com

205
