# Supplementary material for: KSHV infection of B cells primes protective T cell responses in humanized mice
Source: Nat Commun. 2024 Jun 6;15:4841. doi: 10.1038/s41467-024-49209-w (PMC11156630; doi:10.1038/s41467-024-49209-w)
Supplement: Supplementary file 3 — Reporting Summary [file 41467_2024_49209_MOESM3_ESM.pdf]

nature portfolio

Corresponding author(s): Christian Münz

Last updated by author(s): Feb 19, 2024

Reporting Summary

Nature Portfolio wishes to improve the reproducibility of the work that we publish. This form provides structure for consistency and transparency in reporting. For further information on Nature Portfolio policies, see our Editorial Policies and the Editorial Policy Checklist.

Statistics

For all statistical analyses, confirm that the following items are present in the figure legend, table legend, main text, or Methods section.

n/a	Confirmed	
☐	☒	The exact sample size (*n*) for each experimental group/condition, given as a discrete number and unit of measurement
☐	☒	A statement on whether measurements were taken from distinct samples or whether the same sample was measured repeatedly
☐	☒	The statistical test(s) used AND whether they are one- or two-sided<br>*Only common tests should be described solely by name; describe more complex techniques in the Methods section.*
☐	☒	A description of all covariates tested
☐	☒	A description of any assumptions or corrections, such as tests of normality and adjustment for multiple comparisons
☐	☒	A full description of the statistical parameters including central tendency (e.g. means) or other basic estimates (e.g. regression coefficient) AND variation (e.g. standard deviation) or associated estimates of uncertainty (e.g. confidence intervals)
☐	☒	For null hypothesis testing, the test statistic (e.g. *F*, *t*, *r*) with confidence intervals, effect sizes, degrees of freedom and *P* value noted<br>*Give P values as exact values whenever suitable.*
☒	☐	For Bayesian analysis, information on the choice of priors and Markov chain Monte Carlo settings
☒	☐	For hierarchical and complex designs, identification of the appropriate level for tests and full reporting of outcomes
☐	☒	Estimates of effect sizes (e.g. Cohen's *d*, Pearson's *r*), indicating how they were calculated

Our web collection on statistics for biologists contains articles on many of the points above.

Software and code

Policy information about availability of computer code

Data collection	CFX-manager software, FlowJo software version 10, MiXCR (version 3.0.13) software pipeline, Phenochart (v1.0), InForm (v2.4.8), and BD FACS Diva (V8.0.2) software
Data analysis	CFX-manager software, FlowJo software version 10, GraphPad Prism 9, R software version 4.1.1., VDJtools version 1.2.1, Phenochart (v1.0) and InForm (v2.4.8) software

For manuscripts utilizing custom algorithms or software that are central to the research but not yet described in published literature, software must be made available to editors and reviewers. We strongly encourage code deposition in a community repository (e.g. GitHub). See the Nature Portfolio guidelines for submitting code & software for further information.

Data

Policy information about availability of data

All manuscripts must include a data availability statement. This statement should provide the following information, where applicable:

- Accession codes, unique identifiers, or web links for publicly available datasets
- A description of any restrictions on data availability
- For clinical datasets or third party data, please ensure that the statement adheres to our policy

TCR Sequencing Data are available under SRA data base, accession code PRJNA1109285 . All other data and derived parameters are found in the Source Data file.

Further information and requests for resources and reagents should be directed to and will be fulfilled by the lead contact, Prof Christian Münz, PhD (christian.muenz@uzh.ch).

Research involving human participants, their data, or biological material

Policy information about studies with human participants or human data. See also policy information about sex, gender (identity/presentation), and sexual orientation and race, ethnicity and racism.

Reporting on sex and gender	N/A
Reporting on race, ethnicity, or other socially relevant groupings	N/A
Population characteristics	N/A
Recruitment	N/A
Ethics oversight	The use of human fetal liver tissue and PBMCs from leucocyte concentrates of the Zurich Blood Bank was approved by the cantonal ethical committee of Zurich, Switzerland (protocol no. KEK-ZH-Nr. 2010-0057 and KEK-StV-Nr.19/08).

Note that full information on the approval of the study protocol must also be provided in the manuscript.

Field-specific reporting

Please select the one below that is the best fit for your research. If you are not sure, read the appropriate sections before making your selection.

☒ Life sciences ☐ Behavioural & social sciences ☐ Ecological, evolutionary & environmental sciences

For a reference copy of the document with all sections, see nature.com/documents/nr-reporting-summary-flat.pdf

Life sciences study design

All studies must disclose on these points even when the disclosure is negative.

Sample size	Sample sizes of individual mouse experiments were determined by the group size calculations included in the license application (Power=0.8, alpha error=0.05 (but corrected for groups to be compared) by F test with ANOVA, as well as availability of animals with sufficient reconstitution (>20% CD45+ in blood) within each cohort.
Data exclusions	Excluded from the study were virus-inoculated mice without detectable EBV BamHI W fragment or KSHV ORF26 DNA, respectively, in blood or spleen, and no positive signal for EBNA-2 or LANA, respectively, in FFPE tissue sections.
Replication	Replicates are indicated in the figure legends. Between 3 to 14 experimental replicates are reported in the main figures.
Randomization	Each experiment included mice reconstituted with HPCs from one single HFL donor and criteria for animal distribution into experimental groups included sex and human immune reconstitution parameters. This study includes data from mice of 19 reconstituted NSG and NSG-A2 cohorts with 264 animals. Experiments comprise a total of 152 female mice and 112 male mice of 19.6 ± 4.9 weeks of age (Mean ± SD; range: 13-35 weeks). The median frequency (and IQR: 25th, 75th percentile) of huCD45+ cells of peripheral blood lymphocytes before infection was 79.1% (67.1%, 85.7%), huCD3+ T cells of huCD45+ lymphocytes: 34.6% (25%, 47.5%), huCD19+ B cells of huCD45+ lymphocytes: 57.1% (41.1%, 66.4%), huCD4+ cells of huCD3+ huCD45+ lymphocytes: 74.6% (68%, 80.1%), huCD8+ cells of huCD3+ huCD45+ lymphocytes: 22.8% (16.9%, 28.2%).
Blinding	Analysis was performed by mutliple investigators of which all but the first authors were blinded in their analysis.

Reporting for specific materials, systems and methods

We require information from authors about some types of materials, experimental systems and methods used in many studies. Here, indicate whether each material, system or method listed is relevant to your study. If you are not sure if a list item applies to your research, read the appropriate section before selecting a response.

Materials & experimental systems

n/a	Involved in the study	
☐	☒	Antibodies
☐	☒	Eukaryotic cell lines
☒	☐	Palaeontology and archaeology
☐	☒	Animals and other organisms
☒	☐	Clinical data
☒	☐	Dual use research of concern
☒	☐	Plants

Methods

n/a	Involved in the study	
☒	☐	ChIP-seq
☐	☒	Flow cytometry
☒	☐	MRI-based neuroimaging

Antibodies

Antibodies used	See key resources table of the manuscript.
Validation	Antibodies and dilutions were validated by FMO stainings and titration stainings.

Eukaryotic cell lines

Policy information about cell lines and Sex and Gender in Research

Cell line source(s)	Brk.219 (Kati et al 2013), Raji cells (from ATCC), HEK293T (ATCC)
Authentication	See key resources table of the manuscript. In general, cell lines not derived from commercial vendors (eg cell lines derived from infected animals) were checked for surface marker expression to verify cell type (B/T cell), and qPCR was used to confirm infection status.
Mycoplasma contamination	Cell lines were monthly tested for mycoplasma contamination with a commercial PCR test.
Commonly misidentified lines (See ICLAC register)	No commonly misidentified lines were used.

Animals and other research organisms

Policy information about studies involving animals; ARRIVE guidelines recommended for reporting animal research, and Sex and Gender in Research

Laboratory animals	NOD-scid γc-/- mice (NOD.Cg-Prkdcscid Il2rgtm1Wjl/SzJ or NSG) and HLA-A2 transgenic NSG (NOD.Cg-Mcph1Tg(HLA-A2.1)1Enge Prkdcscid Il2rgtm1Wjl/SzJ) mice were obtained from the Jackson Laboratories, bred and maintained at the Institute of Experimental Immunology, University of Zurich. Age of NSG and of NSG-A2 mice: human CD34+ HSC injection in the first week after birth, human immune system reconstitution for 3 months and EBV and/or KSHV infection for four weeks.
Wild animals	No wild animals were used.
Reporting on sex	This study includes data from mice of 19 reconstituted NSG and NSG-A2 cohorts with 264 animals. Experiments comprise a total of 152 female mice and 112 male mice of 19.6 ± 4.9 weeks of age (Mean ± SD; range: 13-35 weeks).
Field-collected samples	No field collected samples were used.
Ethics oversight	All described animal experimentation was reviewed and approved by the veterinary office of the canton of Zurich, Switzerland (116/2008, 148/2011, 209/2014, 159/2017, 212/2022, 213/2022).

Note that full information on the approval of the study protocol must also be provided in the manuscript.

Plants

Seed stocks	N/A
Novel plant genotypes	N/A
Authentication	N/A

Flow Cytometry

Plots

Confirm that:

☒ The axis labels state the marker and fluorochrome used (e.g. CD4-FITC).

☒ The axis scales are clearly visible. Include numbers along axes only for bottom left plot of group (a 'group' is an analysis of identical markers).

☒ All plots are contour plots with outliers or pseudocolor plots.

☒ A numerical value for number of cells or percentage (with statistics) is provided.

Methodology

Sample preparation	White blood cell counts in whole blood were determined with a hematocytometer (Beckman Coulter AcT Diff Analyzer) and subjected to erythrocyte lysis by ACK lysis buffer. Spleens were manually dissociated and filtered through a 70-µm cell strainer, followed by separation of mononuclear cells on Ficoll-Paque gradients by centrifugation at 1000 g for 25 min at room temperature (RT) (GE Healthcare). Samples were stained in 96-well v-bottom plates.
Instrument	BD FACS Canto II, BD LSR II Fortessa and BD FACSymphony A5
Software	BD FACSDiva (8.0.2), FlowJo software version 10, GraphPad Prism 9, R software (version 4.1.1.)
Cell population abundance	Purity of isolated B cells was always above 90%.
Gating strategy	Gating strategies are shown in supplementary figures 2, 5 and 13.

☒ Tick this box to confirm that a figure exemplifying the gating strategy is provided in the Supplementary Information.
